# Seed: a user-friendly tool for exploring and visualizing microbial community data

**DOI:** 10.1093/bioinformatics/btu693

**Published:** 2014-10-20

**Authors:** Daniel Beck, Christopher Dennis, James A. Foster

**Affiliations:** Department of Biological Sciences, University of Idaho, Moscow, ID 83844, USA

## Abstract

**Summary**: In this article we present Simple Exploration of Ecological Data (Seed), a data exploration tool for microbial communities. Seed is written in R using the Shiny library. This provides access to powerful R-based functions and libraries through a simple user interface. Seed allows users to explore ecological datasets using principal coordinate analyses, scatter plots, bar plots, hierarchal clustering and heatmaps.

**Availability and implementation**: Seed is open source and available at https://github.com/danlbek/Seed.

**Contact**: danlbek@gmail.com

**Supplementary information:** Supplementary data are available at Bioinformatics online.

## 1 INTRODUCTION

The proliferation of microbial community profiling is allowing researchers to study microbial communities in new ways. Increasingly, researchers in diverse fields are asking questions relating to how microbial communities vary across samples. For example, researchers studying the human microbiome are interested in how microbial composition changes across body sites and through time (HMP Consortium *et al.*, 2012). Researchers studying disease look at how microbial communities differ between samples from healthy and unhealthy individuals (Srinivasan and Fredricks, 2009). It is now standard practice to use cultivation independent high-throughput sequencing to identify the microbial composition of many samples. This produces a wealth of data about microbial composition in many different environments and conditions. 

In conjunction with advances in sequencing resources, researchers have developed a number of powerful software tools to analyze and visualize this wealth of data. Packages such as mothur (Schloss *et al.*, 2009) and Qiime (Caporaso *et al.*, 2010) aggregate many tools to allow researchers to quickly and efficiently process large sequencing datasets. These currently available packages excel at performing robust, computationally intensive calculations that attempt to minimize the effects of noise and sequencing artifacts on downstream analyses. They often use a non-visual interface for analysis, even when they provide a graphical user interface for their own functions, requiring the user to know specific command and parameter combinations. While this setup is ideal for pipeline development, it is often a hindrance for data exploration. 

Simple Exploration of Ecological Data (Seed) fills a currently unmet need for a tool that allows researchers to quickly and easily visualize and explore the data that results from these pipelines. This so-called exploratory data analysis has an ‘important place in the toolbox of ecologists’ (Borcard *et al.*, 2011). Though there are texts that recommend specific exploratory techniques (Borcard *et al.*, 2011; Legendre and Legendre, 2012), we know of no tool such as Seed that bundles appropriate tools into an easy-to-use system for non-programmers.

In this article, we present Seed, a software package that focuses on data exploration and visualization of microbial community data derived from high-throughput sequencing.

## 2 SEED SOFTWARE

Seed is an open-source application that allows researchers to visually explore microbial community data. It is designed to allow many different analyses and visualizations including principal component and coordinate analysis (PCA/PCoA), hierarchal clustering, scatter plots, bar plots and heatmaps. These plots allow users to visualize similarities and differences among samples and how environmental and microbial features vary across samples.

Seed is written in the R programming language (R Core Team, 2013) using the Shiny framework (RStudio Inc., 2013). R is open source and available for Linux, MacOS and Windows operating systems. The use of R allows us to take advantage of the wealth of R packages available for complex analyses and visualizations.

**Fig. 1.**
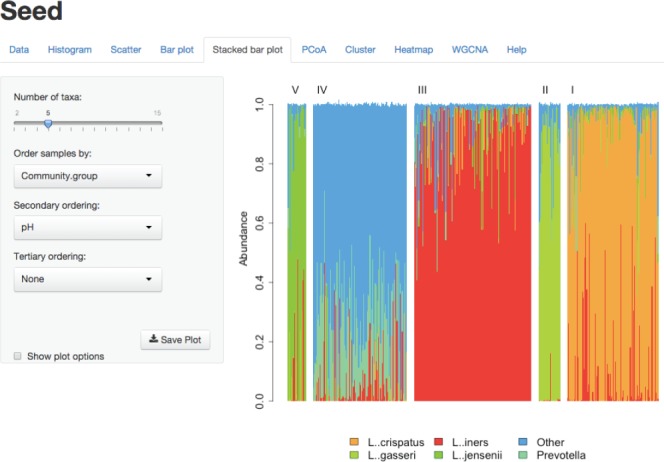
This figure shows Seed’s simple web-based interface. The stacked bar plot shown here is based on data originally published by Ravel *et al.*

Seed is a web-based application, which may be installed locally or hosted on a remote server. When running Seed from a central server, users can access it through a web browser and are not required to install it locally. This means non-expert users can quickly and easily begin using Seed, even without local installations of R. Additionally, updates to R, Shiny, Seed and underlying packages can be done seamlessly and invisibly to the end user. The use of a web browser also provides a familiar interface to most users, allowing them to quickly and easily learn to use Seed. The user interface for seed can be seen in Figure 1.

Currently, Seed requires two types of data, microbial abundance data and sample metadata. The microbial abundance data contain counts or abundances of each microbial taxon in each sample. The sample metadata contain information about each sample, for example the sample pH or temperature. Seed allows the user to modify the abundance data using a number of common transformations including presence/absence, relative abundance and Hellinger transformations. Seed is not limited to microbial data, though that was our primary research domain. It can be used to explore any data that include both feature counts and values for response variables.

Once the user has imported and verified their dataset, they may easily explore their data with many plot types. Examples of some of the plots generated by Seed are shown in the supplementary information. Many of the plots include options to incorporate sample information by coloring points or bars according to metadata values. This allows users to easily visualize the relationship between the sample metadata and the structure of the microbial communities present in the samples.

The design of Seed emphasizes simplicity over exhaustive inclusion of parameters. In many or most cases, researchers will use Seed to understand general trends in the data, which may then inform more specialized analyses. Seed is designed to quickly explore ecological datasets and to act as a hypothesis-generating tool. Publication quality figures and polished analyses are beyond the current scope of this project, though Seed can output all plots in pdf or png format. Additionally, large dataset analysis may be too slow for a comfortable user experience. Note, however, that we used published microbiome and patient data with nearly 400 samples and 250 taxa (Ravel *et al.*, 2011) on a standard laptop while preparing this publication. Seed is certainly capable of handling datasets with hundreds of samples and more than a thousand taxa.

As with any software package, not all analyses have been implemented in Seed. We encourage users to also consider other visualization tools including phyloseq (McMurdie and Holmes, 2013) for analyses incorporating phylogenetic relationships and EMPeror (Vázquez-Baeza *et al.*, 2013) for PCoA analyses of very large datasets. Additionally, while Seed provides some guidance for users, tool selection and result interpretation still relies on user expertise.

## 3 FUTURE DIRECTIONS

Seed is freely available at https://github.com/danlbek/Seed. Development of Seed is ongoing. We are continuing to add new visualizations and to improve existing ones. Future development will focus on adding phylogenetic and taxonomic data structures, which will allow for analyses that take microbial relationships into account. We welcome user contributions to the project and encourage labs to copy and modify the code to suit their own needs.

## Supplementary Material

Supplementary Data
