# Supplementary material for: Seed: a user-friendly tool for exploring and visualizing microbial community data
Source: Bioinformatics. 2014 Oct 20;31(4):602–3. doi: 10.1093/bioinformatics/btu693 (PMC4325548; doi:10.1093/bioinformatics/btu693)
Supplement: Supplementary Data [file supp_31_4_602__index.html]

Seed: a user-friendly tool for exploring and visualizing microbial community data — Seed: a user-friendly tool for exploring and visualizing microbial community data — Seed: a user-friendly tool for exploring and visualizing microbial community data — Supplementary Data 

# Seed: a user-friendly tool for exploring and visualizing microbial community data

## Supplementary Data

files

**Files in this Data Supplement:**

- Supplementary Data - pdf file
