# Supplementary material for: Seed: a user-friendly tool for exploring and visualizing microbial community data
Source: Bioinformatics. 2014 Oct 20;31(4):602–3. doi: 10.1093/bioinformatics/btu693 (PMC4325548; doi:10.1093/bioinformatics/btu693)
Supplement: Supplementary Data [file supp_btu693_walkthrough.pdf]

Seed example analysis:
The vagina microbiome

In this walkthrough, we use data from Ravel *et al.* 2011[1] to demonstrate the utility of Seed. This study sampled the vagina microbiome of nearly 400 women at a single point in time. Additional information was also collected, including pH and the woman's ethnicity. The authors published the data along with the paper. Both are available online through PNAS. This walkthrough is not intended to be a comprehensive list of Seed features, but rather a helpful starting point for analyzing new datasets.

Background

The vagina microbiome is composed of hundreds of different microbe types. It varies widely between women and over time. Researchers study the vagina microbiome for a wide variety of reasons, one of which is the apparent association between the microbiome and bacterial vaginosis (BV). BV is a disease characterized by symptoms including odor, discharge, and irritation. It is widely prevalent, with estimates of affected women as high as 30%. The cause of BV is unknown, however it has been linked with the vagina microbiome. BV is often diagnosed using a metric called the Nugent score. The Nugent score is based on cell morphology and can range between 0 and 10. A Nugent score of 7 or greater is considered consistent with BV.

Data import

To start exploring the dataset, we first load the data files. The data should be in two separate files. The metadata file should include sample information. The taxa file should contain the abundance data. The first column in each file must be the sample names. Seed uses these names to automatically associate sample information with abundance information. Seed expects CSV files by default, however, the user can use the "Show file options" selection to import tab or semicolon separated value files.

After selecting the data files, the user should verify that Seed has read them correctly. Seed displays the first five lines of each data table (Figure 1) as well as the number of samples successfully detected in both the metadata file and the sample file. Seed also lists the dimensions of the raw and processed data files. The user

---

[1] Ravel J, Gajer P, Abdo Z, Schneider M, *et al.* (2011) Vaginal microbiome of reproductive-age women. PNAS 108:4680-4687

should use this information to determine whether the data has been imported correctly.

The abundance data can be transformed to allow for certain analyses. If the raw data is in read counts, it should often be converted to relative abundance in order to make the samples comparable. The abundance data may also be converted to presence/absence which simply measures whether the taxa was detected or not in any particular sample. Finally, the Hellinger transformation may be applied to the data. Further information on ecological data transformations may be found from a variety of sources including Legendre and Gallagher, 2001[2].

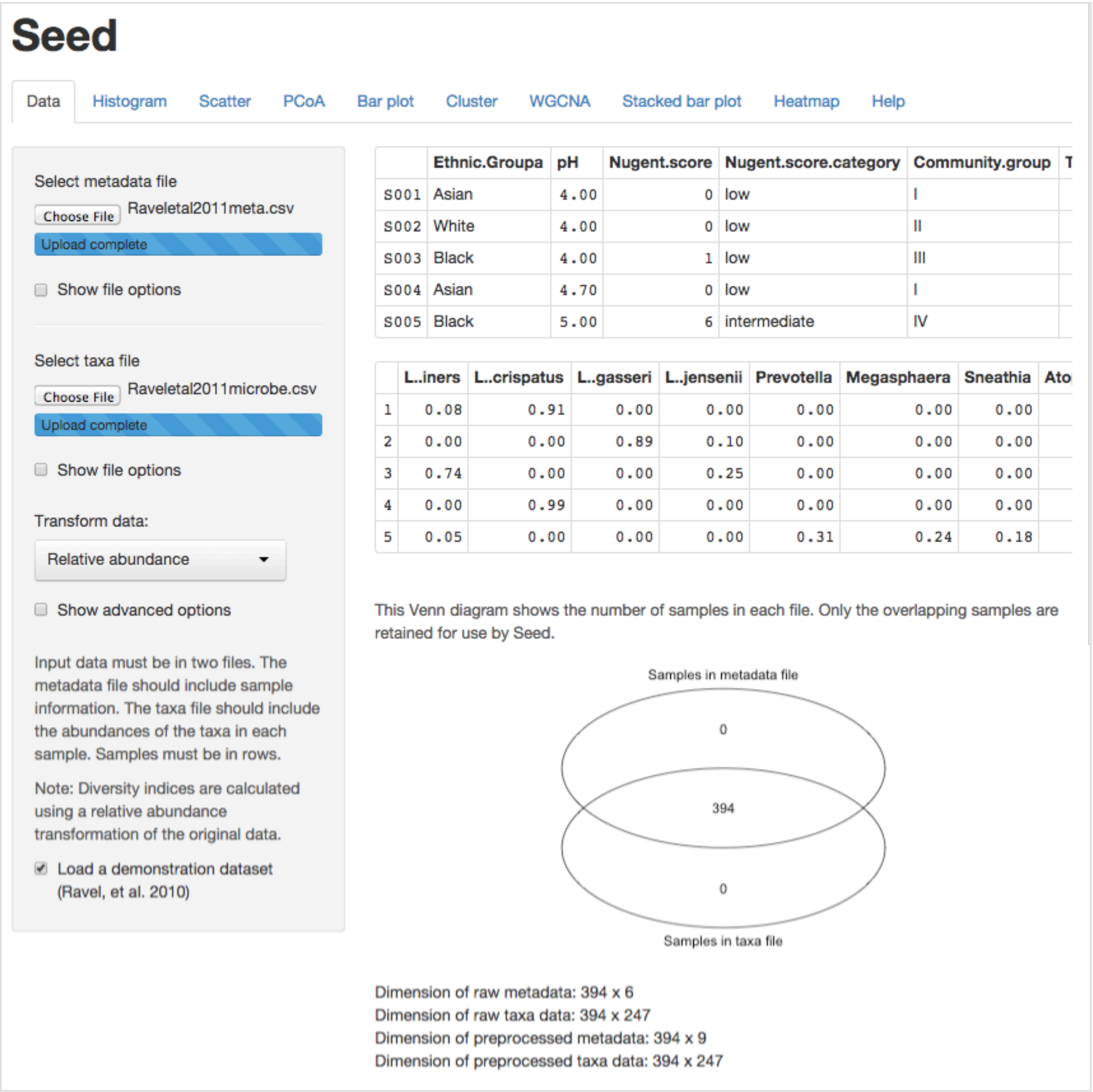

2 Legendre P, Gallagher ED. (2001) Ecologically meaningful transformations for ordination of species data. Oecologia 129:271-280

Figure 1. This figure shows the Seed interface. The user can verify that the data files have loaded correctly using the displayed tables. The Venn diagram can be used to determine whether the sample information has been correctly associated with the abundance data.

Other available Seed features may be accessed using the "Show advanced options" selection.

Histograms

The first data visualization in Seed is the histogram. Histograms show how variable values are distributed across samples. They may be used to answer a number of potentially interesting questions. In the case of the Ravel *et al.* dataset, we may be interested in how microbial taxa vary across samples. For example, various Lactobacillus species are thought to play important rolls in the vagina microbiome. The histogram of L. iners shown in Figure 2 indicates that there are numerous samples with either very high or very low levels of L. iners, however, there are fewer samples with moderate levels of L iners. These patterns may reflect underlying ecological dynamics.

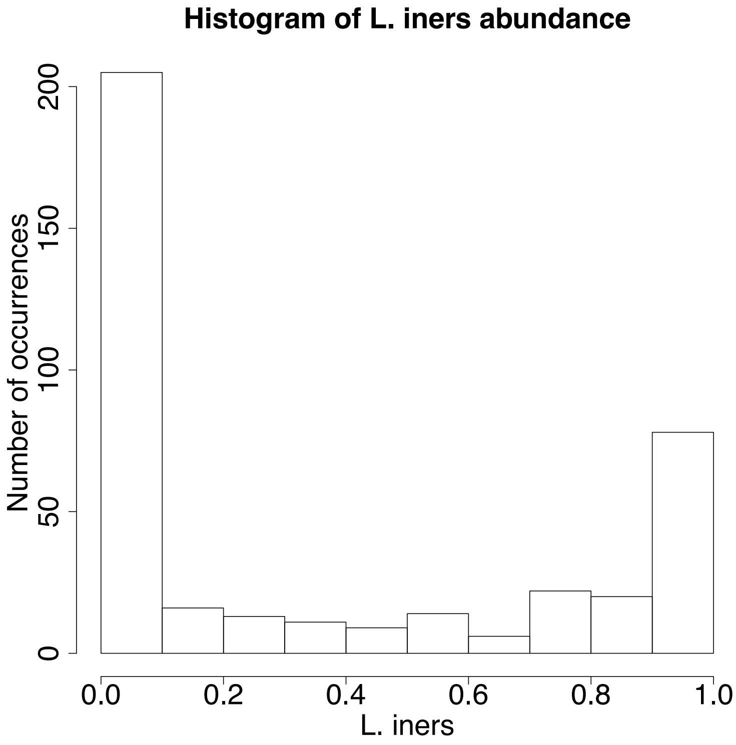

Figure 2. This histogram shows the frequency of L. iners across samples.

Histograms may also be used to verify dataset attributes and to determine how sample metadata is distributed across samples. For example, a histogram of pH values would show whether samples tended to have high, moderate, or low pH.

Users are able to select the number of "Breaks" present in the histogram. A higher number of breaks will increase the number of bars on the histogram. For all plots, Seed allows for some fine scale manipulation of plot options. These features can be accessed using the "Show plot options" selection at the bottom of each visualization sidebar. Common adjustments include axes labels, margin widths, and font sizes.

Scatter plots

Scatter plots can help visualize the relationships between different taxa or show how a taxon varies with sample characteristics. Figure 3 shows the Ravel *et al*. samples plotted by the abundance of L. crispatus and L. iners.

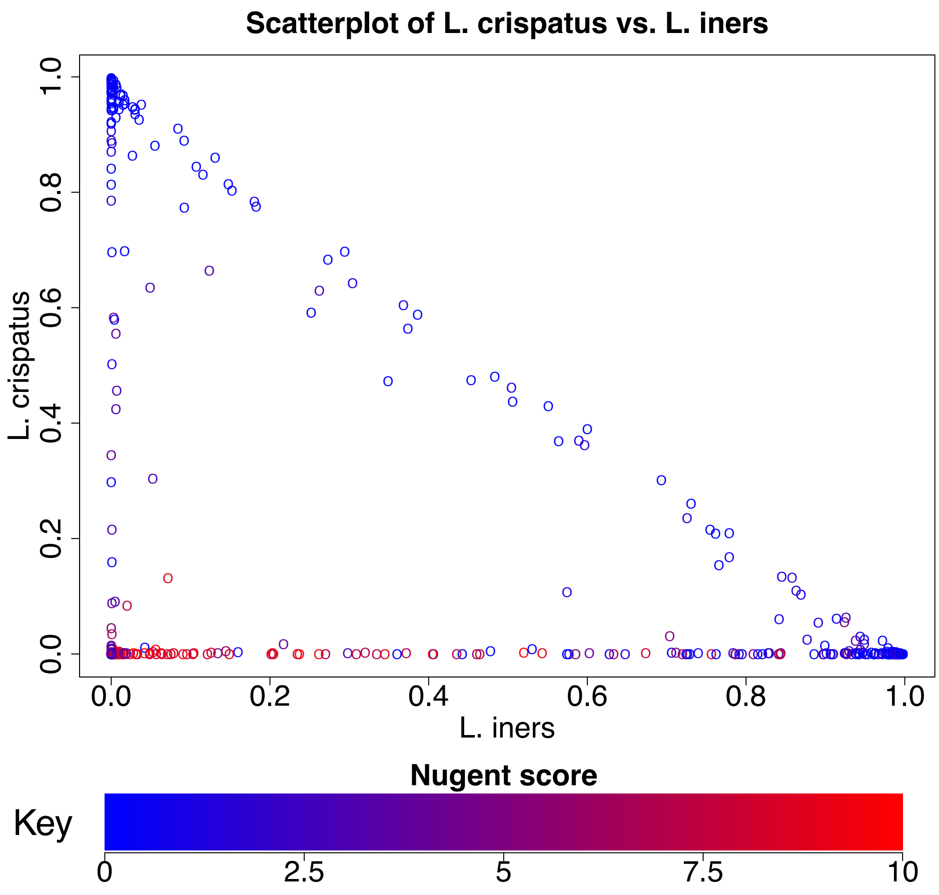

Figure 3. This scatter plot shows each sample plotted by the abundance of L. crispatus and L. iners. The color of the points reflects the Nugent score of the sample. The samples with high abundance of either L. iners or L. crispatus seem to generally have low Nugent scores. Additionally, the distribution of points appears oddly triangular. This may be an interesting pattern to further pursue with targeted analyses.

As can be seen in Figure 3, color can be used to add another dimension to many plots. Seed allows the user to easily color points using sample information. Several color options are available. The "Unique" option is often ideal for categorical data. This option assigns a different color for each variable value (i.e. Figure 4). The "Gradient" option assigns a shade of blue or red to each point, with the smallest value point assigned a blue color, the highest value point a red color, and the rest of the points assigned a color along the blue/red gradient (i.e. Figure 3). The "Categories" option can be used to break a continuous value into groups, each of which are assigned a unique color. This option is useful for separating continuous values into high, medium, and low categories.

As with any analysis tool, users should use caution when generating and interpreting the visualization results. Figure 4 shows a scatterplot of pH vs. ethnic group. In the context of the Ravel *et al.* study, this plot is largely uninteresting and obviously misleading. There are samples from women in each ethnic group at nearly every available pH level. A scatter plot is a bad choice for representing the categorical ethnic group variable. However, it should be emphasized that Seed does not check for unwise plotting decisions and interpretations.

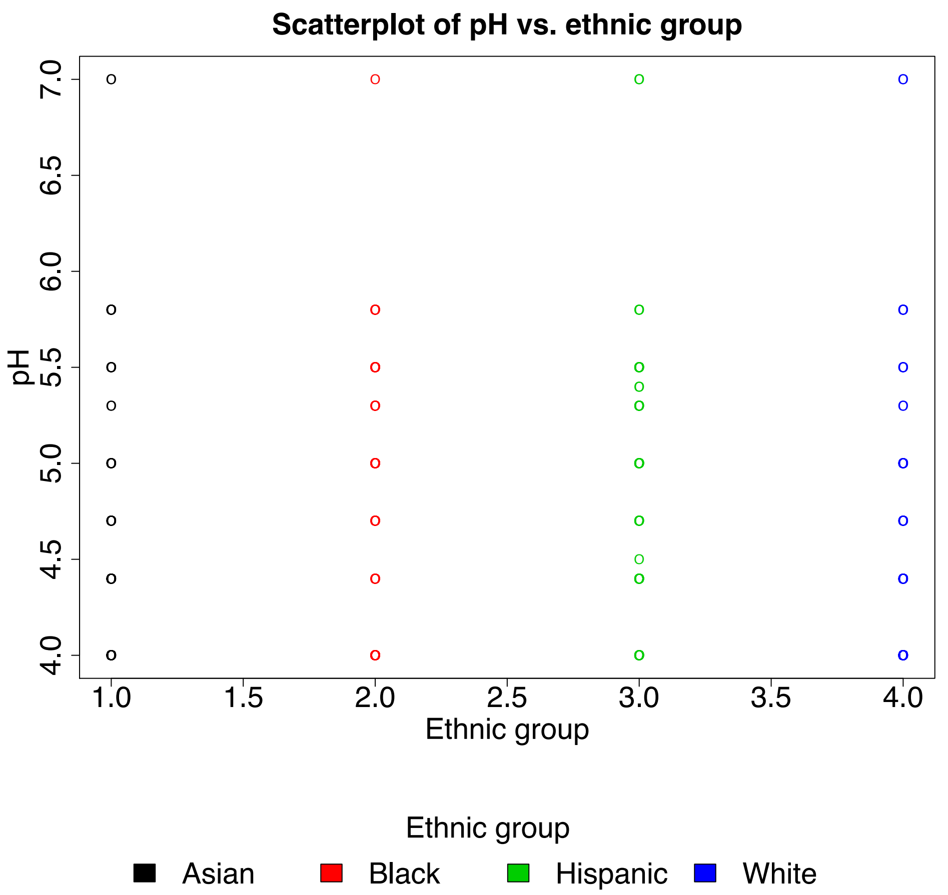

Figure 4. This is an example of a bad scatterplot. Seed is certainly capable of generating plots that may distort underlying patterns and mislead the user. This plot, for example, has many overlapping data points, which obscure any differences between ethnic groups.

Bar plot

Bar plots are often useful visualizations for categorical variables. We can use them to determine whether certain groups are different from other groups on average. For example, in Figure 5 we plot the mean pH in each different ethnic group. The blue numbers in each bar show the number of samples in each group. The error bars show 95% confidence intervals assuming normality and independence.

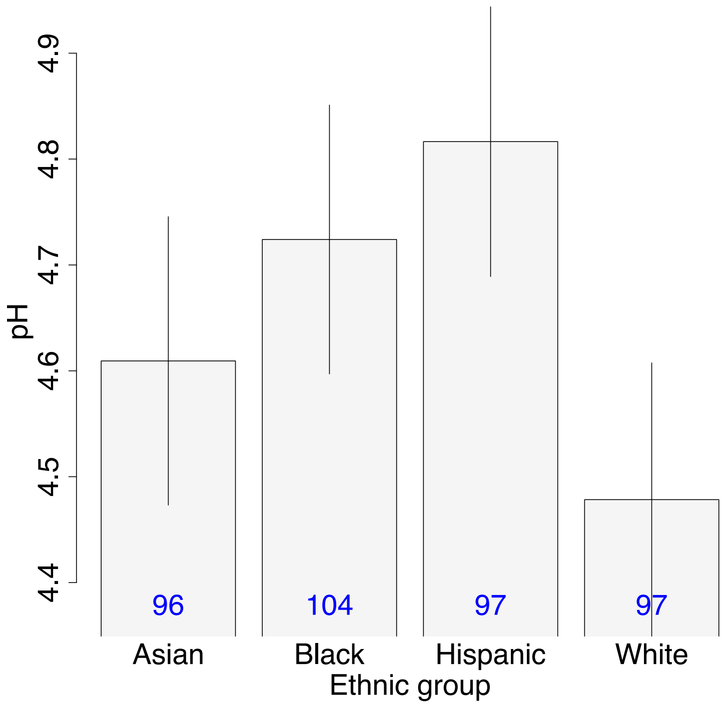

Figure 5. This figure plots the average sample pH across ethnic groups. The blue numbers show how many samples are in each group. Error bars are 95% confidence intervals (assuming normality and independence). As can be seen, there appear to be small differences in pH between White and Hispanic ethnic groups. However, it should be noted that these results are not corrected for multiple tests.

Some variables are not immediately acceptable for bar plot visualizations. For example, plotting L. iners as the bar variable and Nugent score as the value variable results in an unreadable bar plot (Figure 6). Many continuous variables will generally produce absurd plots. However, interesting results may be hidden in these variables. In the case of L. iners, it may be interesting to determine how Nugent

score varies in samples with low, moderate, and high L. iners abundance levels. Seed allows users to transform continuous variables into categories. This is accomplished by selecting the “Categorize bar variable” option. Figure 7 shows L. iners grouped into three separate categories. Samples with low and high L. iners appear to have significantly different Nugent scores.

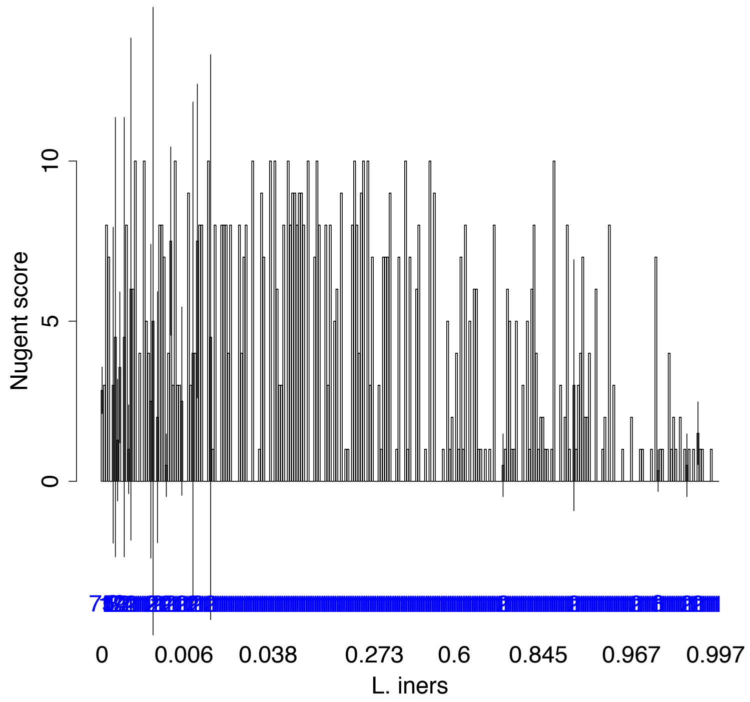

Figure 6. This figure uses a continuous variable (L. iners) as the bar variable, resulting in an unhelpful plot. Seed allows users to break continuous variables into categories, which results in more interpretable plots (i.e. Figure 7).

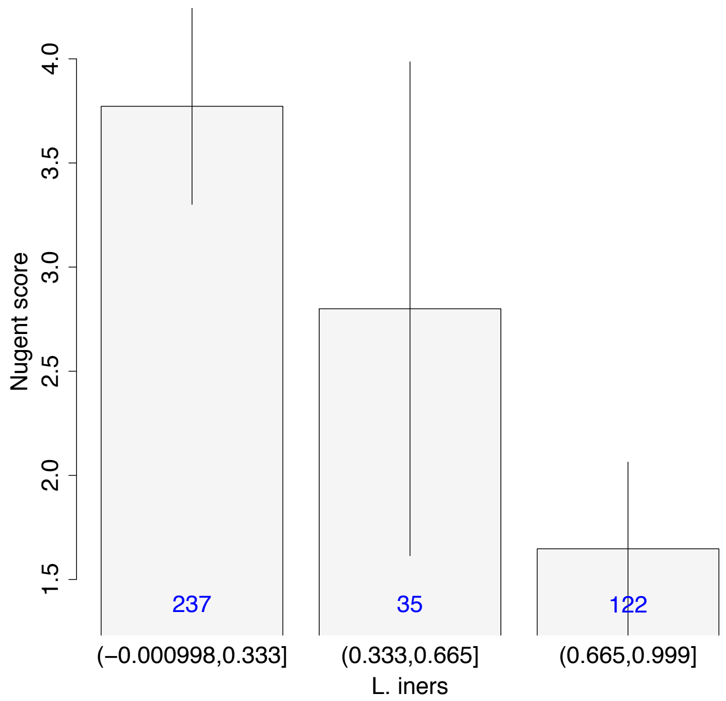

Figure 7. Continuous variables can be divided into evenly spaced groups. Samples with high and low levels of L. iners appear to have significantly different Nugent scores on average. However, the number of categories is user defined and multiple testing may lead to overconfidence in the results.

Seed currently shows no plot when the selected value variable is categorical. It is not meaningful to calculate the average of a variable such as ethnic group. However, Seed also does not yet display a suitable error message for this situation.

Stacked bar plot

Stacked bar plots show each sample as a colored bar. The bar is divided into different colors according to the relative proportion of taxa within the sample. These plots help give an overview of sample composition. These plots can become unreadable quickly when large numbers of different microbes are present. For this reason, only a few most common taxa are generally included. The remaining taxa are lumped together in an "Other" category.

Seed allows users to order samples by up to three different variables. In Figure 8 we group the samples first by community group and second by Shannon diversity. This allows us to see the apparent differences in microbial composition between the community groups.

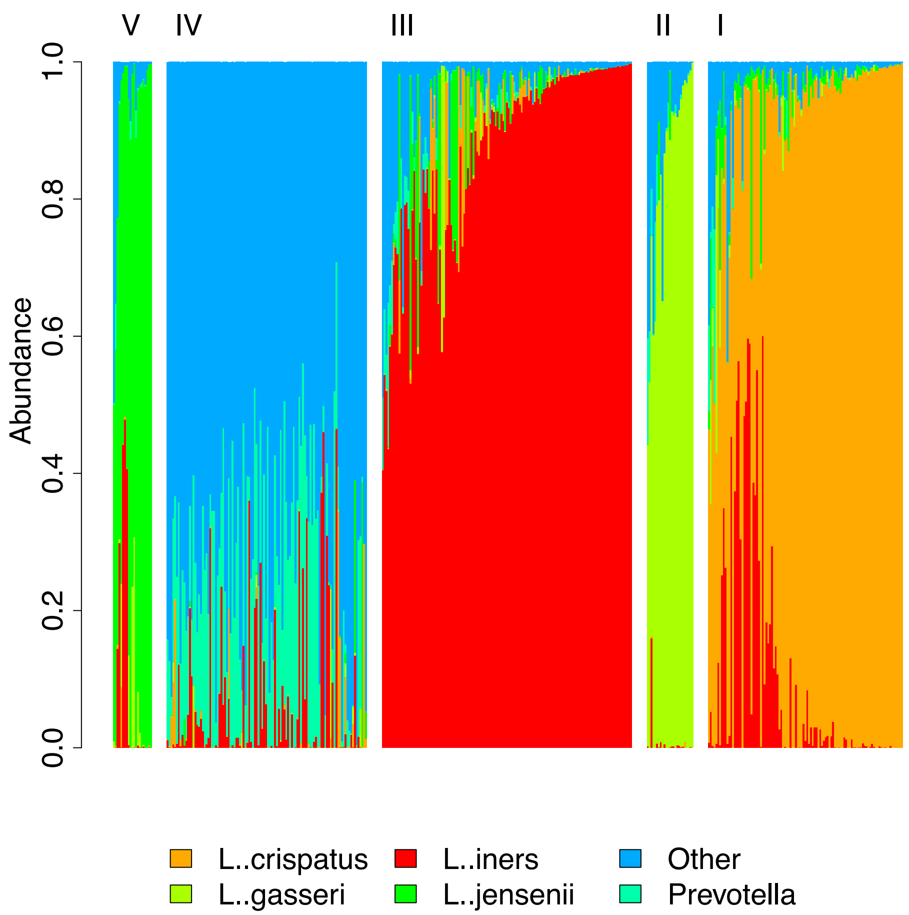

Figure 8. This figure shows the microbial composition of the samples. It is clear that community group IV is composed of many different low-abundance taxa. A single microbe type often dominates samples in the other four groups.

Principal Coordinate Analysis (PCoA)

PCoA plots are useful visualizations for showing how samples relate to each other. Each sample is represented by a number of features. In the case of the ecological datasets, the features are typically the abundances of different taxa. The samples can be compared to each other using several similarity measures. These similarity measures are used to create a distance matrix describing how similar each sample is to every other sample. Samples can then be placed in multidimensional space so that the distance between sample points is proportional to the differences between the samples.

In order to display this information, the points are flattened onto two dimensions. PCoA flattens the points so that the first axis captures as much variation in the sample points as possible. The second axis is chosen to capture as much of the remaining variation as possible. Typically, PCoA plots display these first two axes. However, it is certainly possible to plot other principal coordinate axes. While the

first two axes usually capture most of the sample variation, successive axes may still provide additional insight.

PCoA plots show how samples group together. As can be seen in Figure 9, samples in the Ravel *et al*. dataset appear to cluster into three groups. Extending this visualization to the third principal coordinate axis in Figure 10 reveals the possibility of four groups of points.

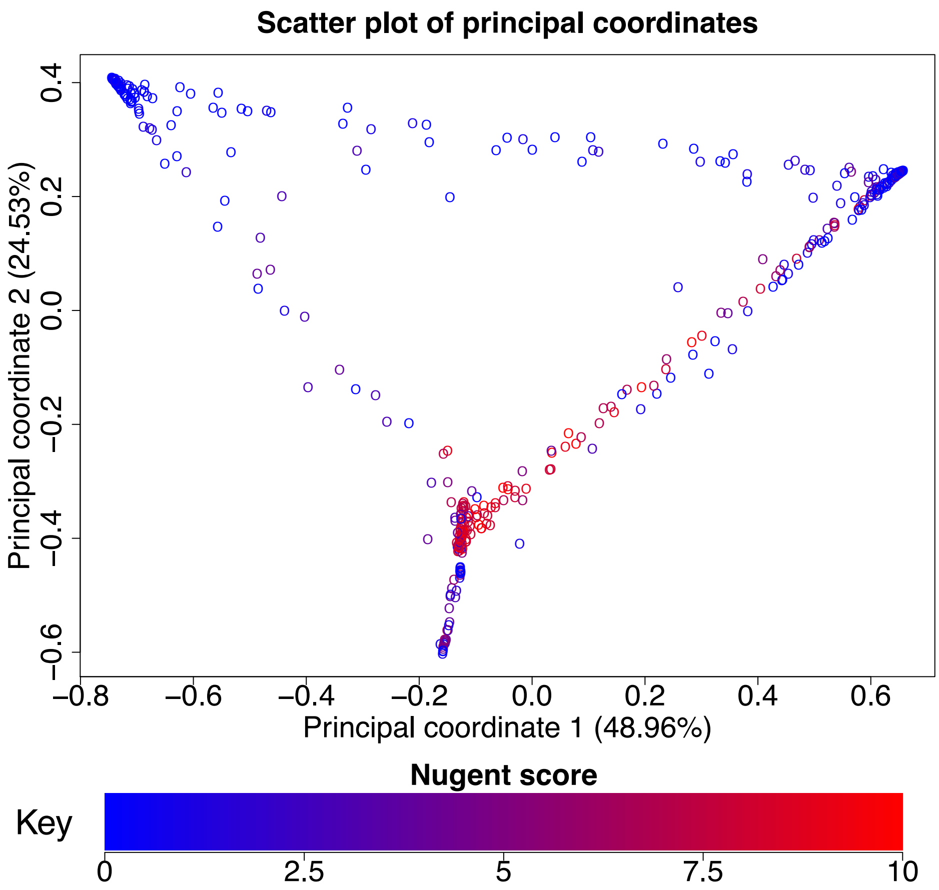

Figure 9. This figure shows the samples plotted by the first two principal coordinate axes. Samples with high Nugent score appear to cluster together.

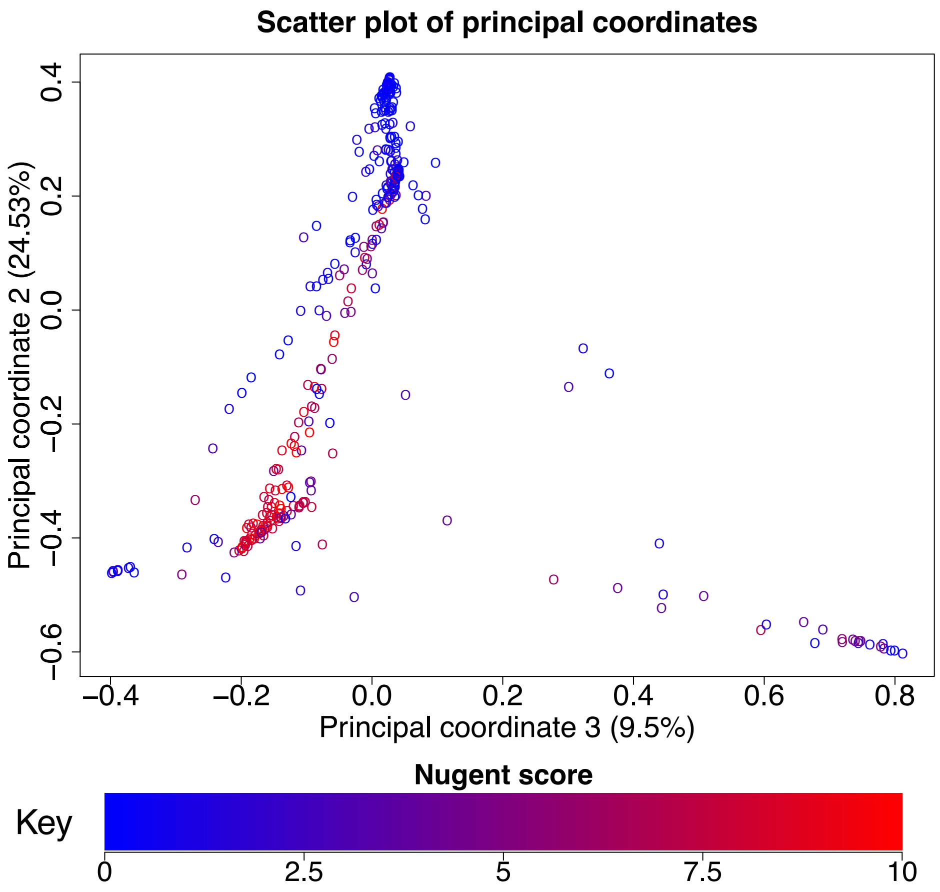

Figure 10. This figure shows the samples plotted by the second and third principal coordinate axes. Using successive axes allows the user to view the points from a different perspective.

Using Seed's color functionality allows us to overlay metadata information onto the plots. This makes it apparent that the high Nugent score samples cluster together. This implies that samples with high Nugent scores have similar microbial composition. It is also clear, however, that some samples with low Nugent scores have similar microbial communities to samples with high Nugent scores. These samples may be candidates for further analysis.

Cluster dendrograms

We can further explore the results of the PCoA analysis using cluster dendrograms. Cluster dendrograms can help visualize whether vaginal microbial communities are well described as belonging to a finite set of community types. Additionally, metadata can be added to these visualizations to determine how community characteristics vary across these groups.

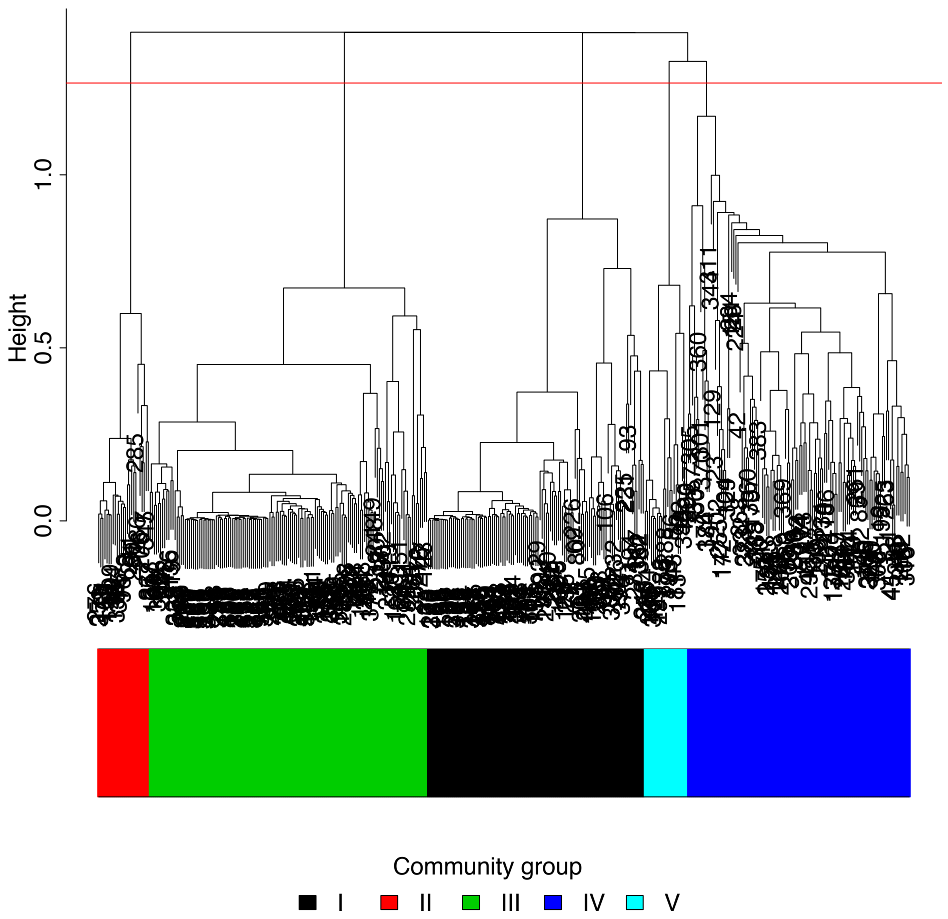

Figure 11. This figure shows the dendrogram produced using Euclidean distances and complete clustering. Different distance and clustering methods may be chosen to suit each dataset.

The user can set the red line shown in Figure 11. Seed uses this line to chop the dendrogram into subtrees. The subtrees can then be explored individually. The color bar below the dendrogram allows the user to link metadata to the samples.

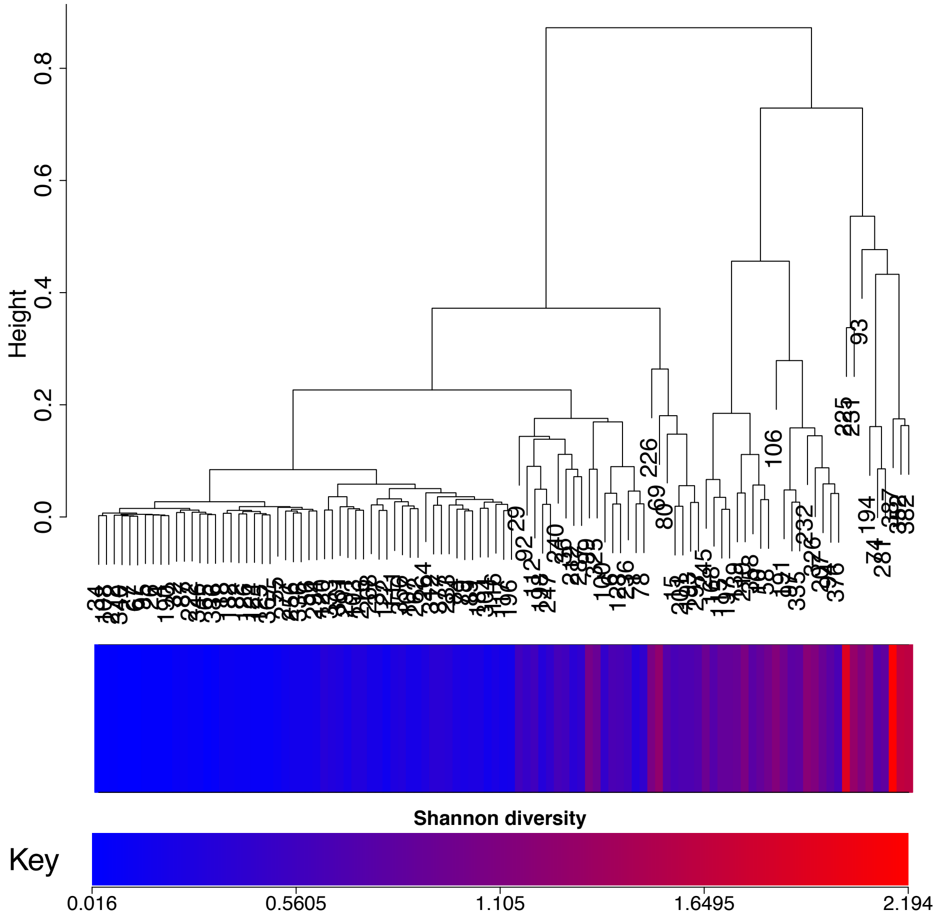

Figure 12. This figure shows the community group I portion of the same cluster dendrogram shown in Figure 11. Users can use Seed to quickly select subtrees of the overall dendrogram for further exploration. In this figure, the color bar is changed to reflect each sample's diversity level.

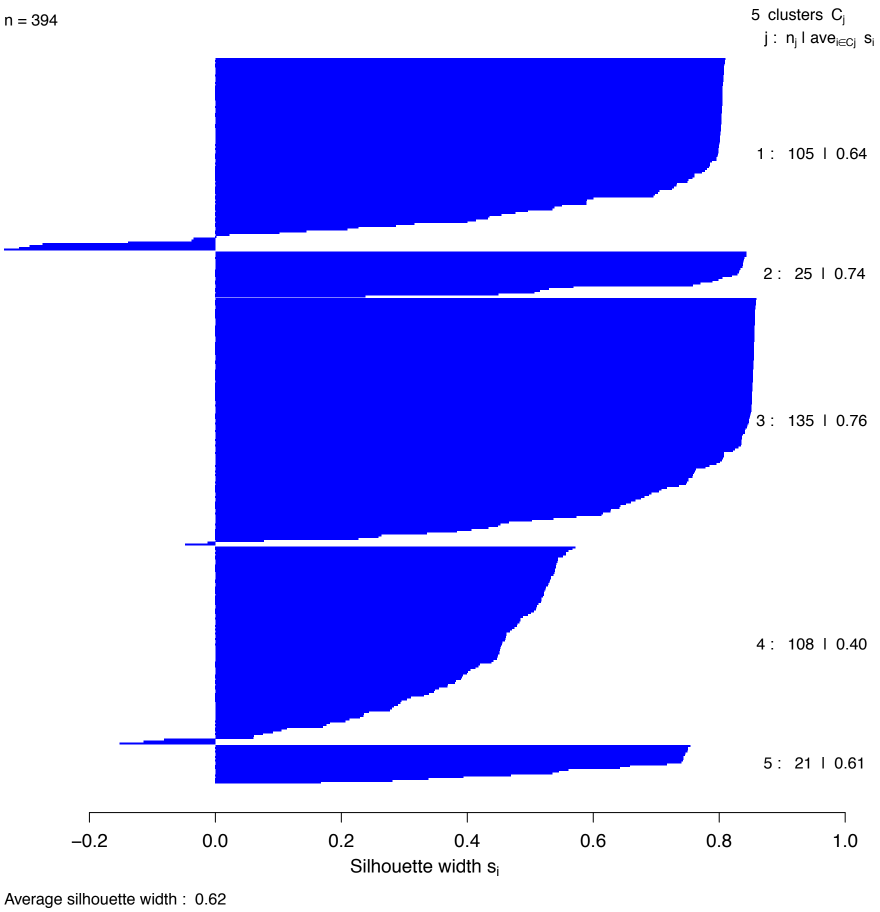

Figure 13. Silhouette plots help validate clusters and estimate the optimal number of clusters in the dataset. In general, a higher average silhouette width indicates better clustering.

Heatmap

Heatmaps combine many individual types of plots and analyses into a single visualization. They are often complex and detailed, providing researchers with a compact way to visualize several different aspects of the dataset. As can be seen in Figure 14, heatmaps can include a clustering dendrogram (at top), abundance information (the red and blue center area), and sample information (lower box). Heatmaps provide an overview of the entire dataset and can summarize substantial amounts of information.

Figure 14 shows the abundance of the most common taxa across samples. The clustering of the samples into discrete community groups is also apparent. The metadata box shows a possible link between diversity and high Nugent score.

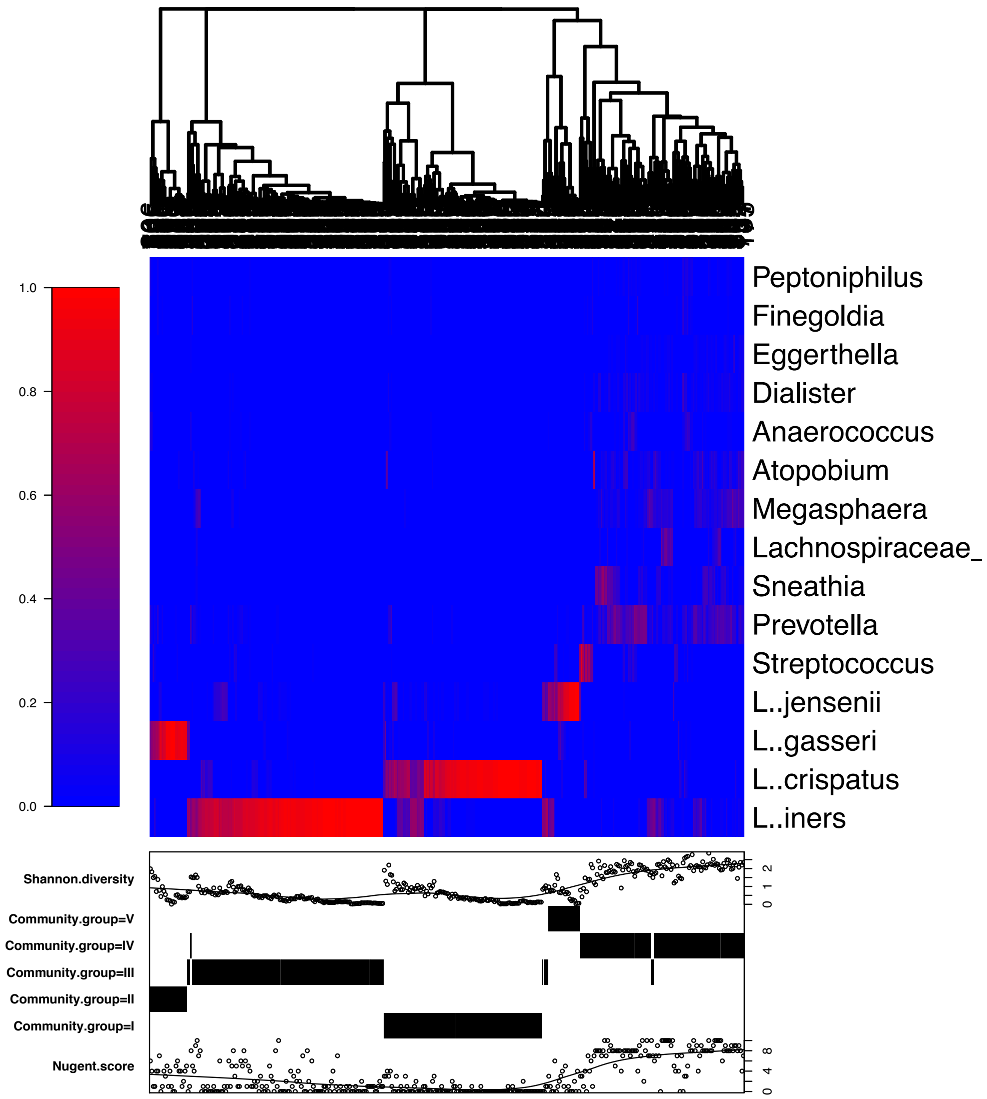

Figure 14. This figure shows a heatmap of the data. It summarizes the abundance levels of the top fifteen most abundant taxa. Here we also show the Nugent score, community group, and Shannon diversity for each sample.

Conclusions

In this walkthrough we have explored many different aspects of the Ravel *et al.* dataset. We used several different plot types, each with advantages and disadvantages. There are many other plots we can use to further explore this dataset. More intricate and targeted analyses may also be necessary to answer specific questions. Seed allows researchers to quickly and easily explore and summarize new ecological datasets. We hope that users will expand and modify Seed to fit their own needs and research goals.
